# A Novel Algorithm for Routing Paths Selection in Mesh-Based Optical Networks-on-Chips

**DOI:** 10.3390/mi11110996

**Published:** 2020-11-09

**Authors:** Xiao-Ping Yang, Ting-Ting Song, Yi-Chen Ye, Bo-Cheng Liu, Hua Yan, Yun-Chao Zhu, Yan-Li Zheng, Yong Liu, Yi-Yuan Xie

**Affiliations:** 1 School of Electronic and Information Engineering, Southwest University, Chongqing 400715, China; xiaoping_415@foxmail.com (X.-P.Y.); ttsong_53@163.com (T.-T.S.); ycye451@swu.edu.cn (Y.-C.Y.); lbc0367@email.swu.edu.cn (B.-C.L.); neil_yanhua@foxmail.com (H.Y.); 15668910386@163.com (Y.-C.Z.); zhengyanlis@email.swu.edu.cn (Y.-L.Z.); 2 School of Optoelectronic Information, University of Electronic Science and Technology of China, Chengdu 611731, China; yongliu@uestc.edu.cn

**Keywords:** optical networks-on-chips, power loss, crosstalk noise, optical signal-to-noise ratio, routing algorithm

## Abstract

Optical networks-on-chips (ONoCs) is an effective and extensible on-chip communication technology, which has the characteristics of high bandwidth, low consumption, and low delay. In the design process of ONoCs, power loss is an important factor for limiting the scalability of ONoCs. Additionally, the optical signal-to-noise ratio (OSNR) is an index to measure the quality of ONoCs. Nowadays, the routing algorithm commonly used in ONoCs is the dimension-order routing algorithm, but the routing paths selected by the algorithm have high power loss and crosstalk noise. In this paper, we propose a 5×5 all-pass optical router model for two-dimensional (2-D) mesh-based ONoCs. Based on the general optical router model and the calculation models of power loss and crosstalk noise, a novel algorithm is proposed in ordder to select the routing paths with the minimum power loss. At the same time, it can ensure that the routing paths have the approximately optimal OSNR. Finally, we employ the Cygnus optical router to verify the proposed routing algorithm. The results show that the algorithm can effectively reduce the power loss and improve the OSNR in the case of network sizes of 5×5 and 6×6. With the increase of the optical network scale, the algorithm can perform better in reducing the power loss and raising the OSNR.

## 1. Introduction

Optical networks-on-chips (ONoCs) is a kind of interconnection mode among multiprocessors on a chip, which adopts optical connection instead of electrical connection [1,2,3]. Moreover, ONoCs has the characteristics of high bandwidth, low consumption, and low delay [4,5]. Accordingly, it is considered to have great application prospects in high-speed communication networks. Power loss and crosstalk noise are the key factors to measure the performance of ONoCs [6]. Hence, it is of great significance to study the power loss and crosstalk noise of ONoCs for information transmission. Nowadays, researchers mainly reduce the power loss and crosstalk noise in ONoCs by constructing new optical network topologies [7,8,9,10,11], designing optical routers [12,13,14,15,16], and using the routing algorithms [17,18,19,20].

By constructing a novel optical network topology, the routing paths can be shortened effectively, thus reducing the power consumption and crosstalk noise of ONoCs. In the design of the optical router structure, the transmission loss and crosstalk can be reduced by reducing the number of microring resonators and waveguide crossings inside the router. Although the performance of ONoCs can be improved by designing new optical network topologies and optical router structures, the reduction of power loss and crosstalk noise of ONoCs becomes decreasingly obvious with the continuous researches on these new structures in recent years. However, on the basis of the new network topologies and router structures, using the routing algorithm can further reduce the power loss and crosstalk noise. Therefore, some researchers begin to study the routing algorithms of the ONoCs in order to find the routing paths with less power loss under the premise of balancing various performances.

Nowadays, the routing algorithm usually used in ONoCs is the dimension-order routing algorithm [21,22,23], but the routing paths that were selected by the algorithm have high power loss and crosstalk noise. In addition, the optimization algorithms are used more in the electrical networks-on-chips (NoCs) [24,25,26,27], but less in the ONoCs. For the use of optimization algorithms in ONoCs, Jae Hoon Lee et al. proposed the insertion loss-aware routing algorithms for fat-tree-based ONoC, which considers the relationship between the insertion loss and routing paths [17]. Marcos A. C. Lima et al. proposed the routing and wavelength assignment method in wavelength division multiplexing (WDM) network based on the genetic algorithm (GA) [18]. In addition, a novel mapping algorithm that combines improved GA and simulated annealing algorithm (SAA) was proposed based on the hybrid optimization strategy [19], by utilizing the algorithm, the optical signal-to-noise ratio (OSNR) of ONoCs can be optimized. Although the use of the traditional GA, SAA, and other algorithms in ONoCs can reduce the power loss to a certain extent, these traditional search algorithms tend to converge earlier in the local sub-optimal solution, whihc leads to premature stagnation in the search. That means they are easy to fall into the local optimal solution, and it is impossible to ensure that the communication path found is the routing path with minimum power consumption.

In this paper, according to the names of optical router ports that we specified, the characteristics of the mesh topology and the routing rules of the optical signal transmission, we propose a novel algorithm for 5×5 all-pass optical routers and mesh networks in order to select the routing paths with minimum power loss. When compared with the dimension-order routing algorithm, the simulation results show that our algorithm can effectively reduce the power loss and improve the OSNR. Meanwhile, the eye diagrams of different routing paths of the mesh ONoCs are discussed.

## 2. Analysis of Power Loss and Crosstalk Noise

**Figure 1:**
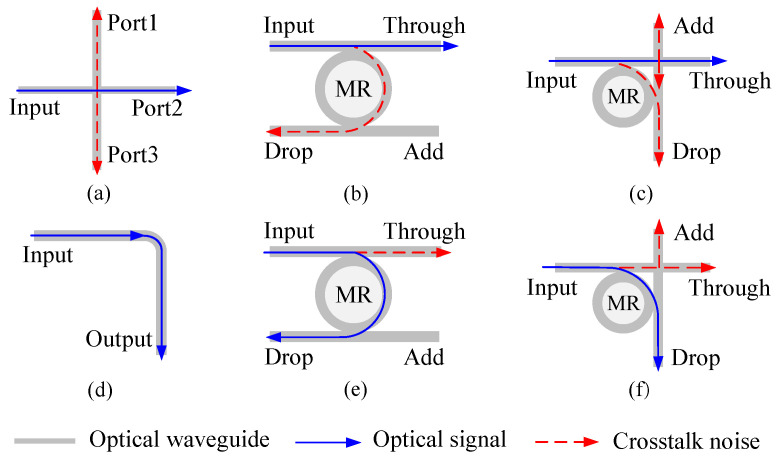
(**a**) Waveguide crossing; (**b**) Parallel switching element (PSE) in OFF state; (**c**) Crossing switching element (CSE) in OFF state; (**d**) Waveguide bend; (**e**) PSE in ON state; (**f**) CSE in ON state.

The basic optical elements consist of waveguides, microring resonators, waveguide bends, waveguide crossings (Figure 1), and so on. Additionally, the most commonly used basic optical switching elements include 1×2 parallel switching element (PSE) and 1×2 crossing switching element (CSE), as shown in Figure 1. Both basic optical switching elements have two states, which are ON state and OFF state, respectively.

### 2.1. Loss Model

When an optical signal is transmitted from one port to another port of the optical router, it will generate power loss, which may include from:

(1) The power loss caused by the waveguide crossings (Figure 1a). When the optical signal enters the waveguide crossing from the Input port, the output power of the Port2 can be calculated by formula (1), where $L_{cr}$ is the crossing loss and $P_{in}$ is the input power.

(2) The power loss caused by the waveguide bends (Figure 1d). When the optical signal passes through a waveguide bend, the corresponding output power can be calculated by formula (2), where $L_{b}$ is the bending loss.

(3) The power loss caused by the basic optical switching elements. As for the 1×2 PSE (Figure 1b,e), it is composed of two optical waveguides and one microring resonator. When the microring resonator is in ON state, it means that the wavelength of the input optical signal is equal to the resonant wavelength of the microring resonator, and then the optical signal is coupled into the microring and transmitted to the Drop port. The output power at the Drop port can be calculated by (3). When the microring resonator is in OFF state, it indicates that the wavelength of the input optical signal is not equal to the resonant wavelength of the microring resonator. Instead of being coupled into the microring, the optical signal is directly transmitted to the Through port. Therefore, the output power of the Through port can be calculated by (4). When compared with PSE, CSE also has two states (Figure 1c,f), but its structure adds a waveguide crossing, so its power loss is different from PSE. When the microring resonator is in ON state, the output power of the Drop port can be calculated by (5), and when it is in OFF state, the output power of the Through port can be calculated by (6), where $L_{pse,on}$ and $L_{pse,off}$ are the insertion loss of each PSE in the ON and OFF states, respectively, $L_{cse,on}$ and $L_{cse,off}$ are the insertion loss of each CSE in the ON and OFF states, respectively.

(1) $$P_{port2}=L_{cr}P_{in}$$

(2) $$P_{out}=L_{b}P_{in}$$

(3) $$P_{D,p,on}=L_{pse,on}P_{in}$$

(4) $$P_{T,p,off}=L_{pse,off}P_{in}$$

(5) $$P_{D,c,on}=L_{cse,on}P_{in}$$

(6) $$P_{T,c,off}=L_{cse,off}P_{in}$$

**Figure 2:**
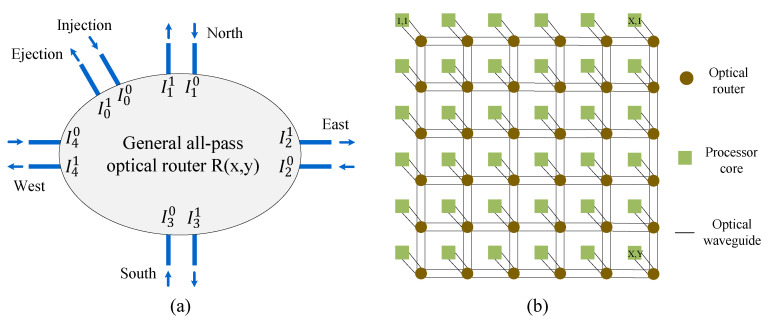
(**a**) General 5×5 optical router model; (**b**) The X×Y 2-D mesh-based ONoC.

Figure 2a shows a general 5×5 all-pass optical router model. The optical router has five bidirectional ports, which are Injection/Ejection, North, East, South, and West. In addition, $I_{n}^{m}$ ($m\in \left\{0,1\right\}$, $n\in \left\{0,1,2,3,4\right\}$) is used in order to describe the flow direction of the optical signal, and 0, 1, 2, 3, and 4 are used to represent the five ports. Besides, we utilize 0, 1 to indicate whether the optical signal flows into or out of the optical router. For example, $I_{1}^{1}$ indicates that an optical signal flows out of the optical router from the North port.

(7) $$\begin{array}{cc} L_{i,j}\left(x,y\right) & =\left\{\begin{array}{cc} S_{i,0}\left(x,y\right)L_{p}^{W_{i,0}\left(x,y\right)} & j=0\\ S_{i,j}\left(x,y\right)L_{p}^{W_{i,j}\left(x,y\right)}L_{p}^{D} & j\neq 0 \end{array}\right.\\ D≅\sqrt{\frac{S}{X\times Y}}\;i,j\in & \left\{0,1,2,3,4\right\},\;x\in \left\{1,2,\ldots ,X\right\},\;y\in \left\{1,2,\ldots ,Y\right\} \end{array}$$

When the optical signal transfers from the *i*th port to the *j*th port of the optical router R(x,y), the insertion loss that is represented by $L_{i,j}\left(x,y\right)$ can be calculated (7). There are two situations need to be considered: First, j=0, namely the output port is the Ejection port, and the corresponding insertion loss can be calculated by (7), in which $S_{i,j}\left(x,y\right)$ denotes the switching power loss that is introduced by the waveguide crossings, the switching elements, and the propagation loss inside the optical router. Second, j≠0, which is the output port is not the Ejection port, so the propagation loss of the optical waveguide between the current router and the next one also should be considered, as shown in (7). *D* is the hop length, which can be calculated by chip size *S* and network size X×Y.

When the optical signal is transmitted from the *i*th port to the *j*th port of the optical router, the optical signal power $P_{out,j}$ can be calculated by (8). $P_{in,i}$ is the power, which injects into the *i*th port of the optical router, and the *x* and *y* represent the abscissa and ordinate of the optical router in an *X*×*Y* 2-D mesh-based ONoCs (Figure 2b), respectively.

(8) $$P_{out,j}=P_{in,i}L_{i,j}\left(x,y\right)\;i,j\in \left\{0,1,2,3,4\right\},x\in \left\{1,2,\ldots ,X\right\},y\in \left\{1,2,\ldots ,Y\right\}$$

Some assumptions are made when we analyze the optical link loss. It is assumed that the power loss of optical signals is the same when they are transmitted from the *i*th port to the *j*th port for different routers in ONoCs, in other words, $L_{i,j}\left(x_{0},y_{0}\right)=L_{i,j}\left(x_{1},y_{1}\right)=L_{i,j}$, $x_{0},x_{1}\in \left\{1,2,\ldots ,X\right\}$, $y_{0},y_{1}\in \left\{1,2,\ldots ,Y\right\}$. These calculation formulas of the power loss mentioned above correspond to the optical router level. At the network level, the power loss of the entire optical link can be calculated by the loss combination of all routers on the link. An *X*×*Y* 2-D mesh-based ONoC, as shown in Figure 2b. When the optical signal travels from the processor core (1, 1) to the processor core (X,Y) by using the dimension-order routing algorithm, it will generate power loss $P_{link((1,1)(X,Y))}$. The power loss of the entire optical link can be defined in (9). The traditional dimension-order routing algorithm, namely the X-Y routing algorithm, is a deterministic routing method. Assuming that all of the optical routers in the ONoCs are represented by a two-dimensional coordinate (x,y), in the way of dimension-order routing algorithm, the optical signal is transmitted first in the X-axis direction, and then transmitted in the Y-axis direction.

(9) $$P_{link((1,1)(X,Y))}=L_{0,2}L_{4,2}^{X-2}L_{4,3}L_{1,3}^{Y-2}L_{1,0}P_{in}$$

### 2.2. Crosstalk Model

When an optical signal travels from one port to another port of the optical router, there may be crosstalk noise inside the router. Similar to the insertion loss analysis, the sources of crosstalk noise can be analyzed from the following aspects:

(1) Crosstalk noise will be produced when two optical signals pass through a waveguide crossing at the same time. When the optical signal enters the waveguide crossing from the Input port, the output power of Port1 and Port3 can be calculated by (10), where $C_{cr}$ is the crosstalk coefficient of the crossing.

(2) The crosstalk noise caused by the basic optical switching elements. As for the 1×2 PSE, when the microring resonator is in ON state, the output power of the Through port can be calculated by (11), where $C_{pse,on}$ is the crosstalk coefficient of each PSE in the ON state. Because the crosstalk noise at the Add port is very small, the crosstalk of the Add port can be ignored. When the microring resonator is in OFF state, the output power of the Drop port can be calculated by Formula (12), where $C_{pse,off}$ is the crosstalk coefficient of each PSE in the OFF state. For the 1×2 CSE, when the microring resonator is in ON state, the output power of the Through port and the Add port can be calculated by (13) and (14), respectively. When the microring resonator is in OFF state, the output power of the Drop port and the Add port can be calculated by (15) and (16), respectively.

(10) $$P_{port1}=P_{port3}=C_{cr}P_{in}$$

(11) $$P_{T,p,on}=C_{pse,on}P_{in}$$

(12) $$P_{D,p,off}=C_{pse,off}P_{in}$$

(13) $$P_{T,c,on}=C_{pse,on}L_{cr}P_{in}$$

(14) $$P_{A,c,on}=C_{pse,on}C_{cr}P_{in}$$

(15) $$P_{D,c,off}=\left(C_{pse,off}+L_{pse,off}^{2}C_{cr}\right)P_{in}$$

(16) $$P_{A,c,off}=L_{pse,off}C_{cr}P_{in}$$

When the optical signal travels from the *i*th port to the *j*th port of the optical router R(x,y), the total crosstalk noise $C_{i,j}\left(x,y\right)$ can be calculated by (17), where $P_{k}\left(x,y\right)$ represents the signal power, which injected into the optical router R(x,y) from the *k*th port, and $K_{i,j,k}\left(x,y\right)$ is the crosstalk noise coefficient of the optical signal that is introduced by $P_{k}\left(x,y\right)$ into the router R(x,y). For the approximate worst-case crosstalk noise model proposed in this paper, we assume that all optical routers are the same, namely, it is assumed that the five ports of the edge routers exist and can be occupied.

(17) $$C_{i,j}\left(x,y\right)=\sum_{k=0}^{4}P_{k}\left(x,y\right)K_{i,j,k}\left(x,y\right)\;i,j,k\in \left\{0,1,2,3,4\right\},x\in \left\{1,2,\ldots ,X\right\},y\in \left\{1,2,\ldots ,Y\right\}$$

OSNR is the ratio of signal power to noise power, and it is a crucial parameter that used to measure the performance of ONoCs. Formula (18) can be used to calculate OSNR, where $P_{\left(x_{0},y_{0}\right),\left(x_{1},y_{1}\right)}$ and $P_{N\left(x_{0},y_{0}\right),\left(x_{1},y_{1}\right)}$ represent the optical signal power and optical noise power of the optical signal transmitted from the optical router $R(x_{0},y_{0})$ to the optical router $R(x_{1},y_{1})$, respectively.

(18) $$OSNR=10\log_{10}\left(\frac{P_{\left(x_{0},y_{0}\right),\left(x_{1},y_{1}\right)}}{P_{N\left(x_{0},y_{0}\right),\left(x_{1},y_{1}\right)}}\right)\;x_{0},x_{1}\in \left\{1,2,\ldots ,X\right\},y_{0},y_{1}\in \left\{1,2,\ldots ,Y\right\}$$

## 3. Minimum-Loss Routing Algorithm

Currently, the graph theory is mainly used in order to optimize the power consumption of ONoCs. In this paper, we put forward a new general method for obtaining the optical link with minimum power loss for the mesh-based ONoCs. When the optical signal travels from core $\left(x_{0},y_{0}\right)$ to core $\left(x_{1},y_{1}\right)$, the optical link goes through fewer optical routers will have better performance in terms of crosstalk noise and time delay. Therefore, the algorithm that is proposed in this paper is based on the premise that the optical link passes through the minimum number of optical routers, which is, $|x_{0}-x_{1}\left|+\right|y_{0}-y_{1}|+1$ optical routers.

We describe the pseudo-code of the algorithm when $x_{0}$≥$x_{1}$ and $y_{0}$≥$y_{1}$, as shown in Algorithm 1. Firstly, we need to judge the relative position of the source node and the destination node. If the two nodes have the same vertical or horizontal coordinates, the optical signal transmission follows the dimension-order routing algorithm, as shown in lines 3–5, and the $Link_{xy}$ represents the optical link selected by using the dimension-order routing algorithm. According to the names of the ports of the optical router and the characteristics of the mesh topology, we can know that there is a relationship between optical links and the ports of the optical router. For example, when the optical signal outputs from the East port of one optical router to the next optical router, the input port must be the West port of the next router.

**Algorithm 1** Routing algorithm for minimum power loss.
**Input:** The source processor core $\left(x_{0},y_{0}\right)$; The destination processor core $\left(x_{1},y_{1}\right)$;**Output:** The optical routing path with the minimum power loss, Link;1: **function**MinimumLossRouting($\left(x_{0},y_{0}\right),\left(x_{1},y_{1}\right)$)2:     Judge the relative position of the core $\left(x_{0},y_{0}\right)$ and the core $\left(x_{1},y_{1}\right)$;3:     **if** $x_{0}=x_{1}$ or $y_{0}=y_{1}$ **then**4:         Adopt the dimension-order routing algorithm;5:         $Link=Link_{xy}$;6:     **else**7:         Generate a sequence containing an Ejection, $|x_{0}-x_{1}|$ West, $|y_{0}-y_{1}|$ North;8:         Initialize an unsorted optical link, Link:link(Injection,α), $link(East,\beta_{1})$, $link(East,\beta_{2})$,…, $link(East,\beta_{|x_{0}-x_{1}|})$, $link(South,\gamma_{1})$, $link(South,\gamma_{2})$, …, $link(South,\gamma_{|y_{0}-y_{1}|})$, $\alpha \in \left\{West,North\right\}$, $\beta ,\gamma \in \left\{Ejection,West,North\right\}$;9:         For the $\left.\{Ejection,|x_{0}-x_{1}|West,|y_{0}-y_{1}|North\right.\}$ sequence, full permutations with repeating elements are produced, these full permutations corresponding to α, β and γ in the Link;10:         Calculate and compare the power loss of these unsorted optical links;11:         Select the Link with the minimum loss;12:         Constraint: comply with the rules of optical signal transmission among the ports of the 5×5 optical routers;13:         Sort the Link and get multiple optical links $Link_{k},k\in \left\{0,1,2\ldots \right\}$;14:         **if** $number_{link_{k}}=1$ **then**15:            Link = $Link_{k}$;16:         **else**17:            Calculate the OSNR of multiple optical links;18:            Select optical link with the optimal OSNR, $Link_{osnr}$, $Link_{osnr}\in Link_{k}$;19:            Link = $Link_{osnr}$;20:         **end if**21:     **end if**22:     **return** Link23: 
**end function**



In order to illustrate the algorithm, we take core $\left(x_{0},y_{0}\right)$ as the source node, core $\left(x_{1},y_{1}\right)$ as the destination node. When the optical signal is transmitted from the initial node to the target node, it passes through the same number of East and West ports, the same number of South and North ports, which are $|x_{0}-x_{1}|$ and $|y_{0}-y_{1}|$, respectively. Besides, it also passes through an Injection port and an Ejection port. Therefore, we generate a sequence that contains an Ejection port, $|x_{0}-x_{1}|$ West ports, and $|y_{0}-y_{1}|$ North ports. This sequence contains all of the output ports of the optical routers from core $\left(x_{0},y_{0}\right)$ to core $\left(x_{1},y_{1}\right)$. Subsequently, we initialize an unsorted optical link: Link, where link(Injection,α) indicates that the optical signal travels from the Injection port to the α port of an optical router. Afterwards, we permute the sequence that has repeating elements to be full permutations, and these full permutations correspond to the α, β, γ ports of the unsorted links. After that, we calculate the power loss of the unsorted links, and select an unsorted link with the minimum power loss, as show in lines 7–11. Finally, in lines 12–20, according to the names of the ports of the optical router and the characteristics of the mesh topology, we sort the unsorted links. For example, when the optical signal travels from core (1, 1) to core (3, 3), the Link: link(Injection,East), link(West,South), link(North,East), link(West,South), link(North,Ejection) is the optical link after sorting. If there is only one optical link after sorting, then this optical link is the selected link with the minimum power loss. If there are multiple optical links, we use formula (18) to calculate their OSNR, and then select the optical link with optimal OSNR as the minimum power loss link. Therefore, the optical link that has the minimum noise power (the one with the approximately optimal OSNR) among the optical links with the equal and minimum loss is chosen to be the final output.

As we know, ($x_{0}$≥$x_{1}$, $y_{0}$≥$y_{1}$), ($x_{0}$≥$x_{1}$, $y_{0}$<$y_{1}$), ($x_{0}$<$x_{1}$, $y_{0}$≥$y_{1}$), ($x_{0}$<$x_{1}$, and $y_{0}$<$y_{1}$) are the four location relationships between the source node and the destination node. In Algorithm 1, we elaborate the pseudo-code of the algorithm when $x_{0}$≥$x_{1}$ and $y_{0}$≥$y_{1}$. For the other three cases, the algorithm idea of their corresponding algorithms is the same as that of Algorithm 1, and the difference among them is that the ports contained in the sequence and the ports contained in the unsorted optical link are different.

## 4. Simulation Results and Discussions

**Figure 3:**
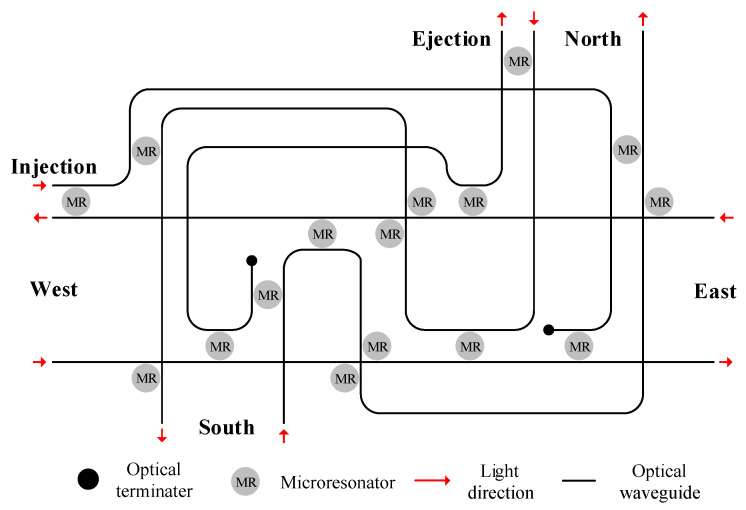
Cygnus optical router.

In this section, we use Gurobi, Python, MATLAB, and Optisystem for the simulation. The Cygnus router is used to construct the 2-D mesh ONoCs [13]. In addition, we choose the traditional dimension-order routing algorithm as a contrast. By comparing the power loss and OSNR between the optical links that were selected by our method and the traditional routing paths, we can verify our algorithm. Besides, in the simulation, the input power is 0 dBm and the other parameters used are shown in Table 1. Figure 3 shows the structure of the Cygnus optical router. It has five bidirectional ports, which are Injection/Ejection, North, East, South, and West. Apart from that, the optical router has a non-blocking feature, so the optical signal can travel from one port to any other port.

In addition, we take processor core (1,1) as the source node. When the optical signals are transmitted from processor core (1,1) to other processor cores, the average loss of the optical links ${\bar{P}}_{link}$ can be calculated by Formula (19). $P_{link((1,1)(x,y))}$ represents the power loss of the optical link when the optical signal travels from core (1, 1) to core (x,y). Moreover, *x* and *y* can not be 1 at the same time.

(19) $${\bar{P}}_{link}=\frac{\sum_{x=1}^{X}\sum_{y=1}^{Y}P_{link((1,1)(x,y))}}{XY-1}$$

**Figure 4:**
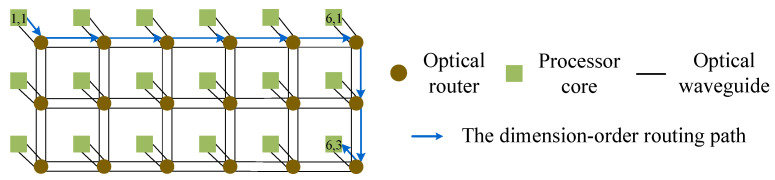
Example: The dimension-order routing path when the optical signal travels from processor core (1, 1) to processor core (6, 3).

Figure 4 shows an example for explaining the dimension-order routing algorithm. There is a pair of communication nodes: core (1, 1) → core (6, 3). Firstly, the optical signal is injected into the optical network through the Injection port of the optical router R(1,1). Subsequently, the optical signal is horizontally transmitted to the optical router R(6,1). The optical signal enters from the West port of the optical router R(6,1), and outputs from its South port. After that, it transmits vertically to the optical router R(6,3). Finally, the optical signal is output from the Ejection port of the optical router R(6,3) and the data are exchanged with the processing core (1,1). It can be seen that the signal power of the optical routing path is equal to $L_{0,2}L_{4,2}^{4}L_{4,3}L_{1,3}L_{1,0}P_{in}$, as shown in Figure 4.

**Figure 5:**
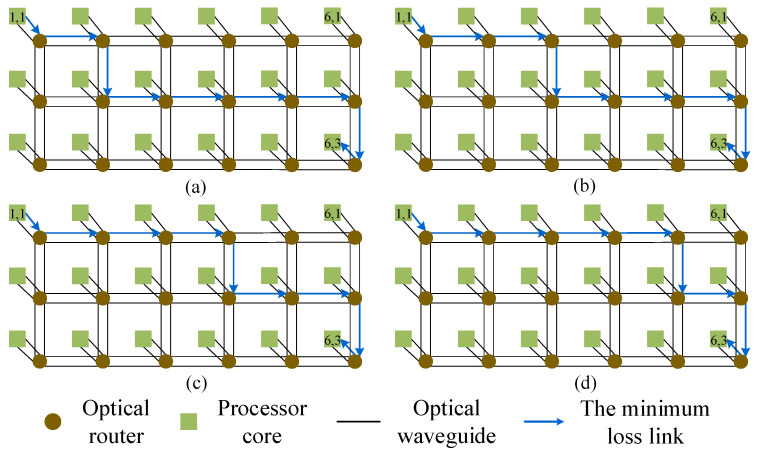
Example: Four routing paths with the minimum power loss when optical signals travel from processor core (1, 1) to processor core (6, 3). (**a**) Link: link(Injection,East), link(West,South), link(North,East), link(West,East), link(West,East), link(West,East), link(West,South), link(North,Ejection); (**b**) Link: link(Injection,East), link(West,East), link(West,South), link(North,East), link(West,East), link(West,East), link(West,South), link(North,Ejection); (**c**) Link: link(Injection,East), link(West,East), link(West,East), link(West,South), link(North,East), link(West,East), link(West,South), link(North,Ejection); (**d**) Link: link(Injection,East), link(West,East), link(West,East), link(West,East), link(West,South), link(North,East), link(West,South), link(North,Ejection).

Figure 5 shows an example to explain our algorithm. There is a pair of communication nodes: core (1, 1) → core (6, 3). Firstly, we generate a sequence containing an Ejection port, |1−6|=5 East ports, |1−3|=2 South ports, then we initialize an unsorted link, Link: link(Injection,α), $link(West,\beta_{1})$, $link(West,\beta_{2})$, $link(West,\beta_{3})$, $link(West,\beta_{4})$, $link(West,\beta_{5})$, $link(North,\gamma_{1})$, $link(North,\gamma_{2})$. Secondly, according to our method, we select out the unsorted link, link(Injection,East), link(West,South), link(West,South), link(West,East), link(West,East), link(West,East), link(North,Ejection), link(North,East), which has the lowest power loss. Next, we sort the optical link and obtain four optical links with different routing paths but the same power loss. As shown in Figure 5, it can be seen that the signal power of the four optical links is equal to $L_{0,2}L_{4,2}^{3}L_{4,3}^{2}L_{1,2}L_{1,0}P_{in}$. Finally, on the basis of formula (18), we select the optimal optical link, as shown in Figure 5d.

**Figure 6:**
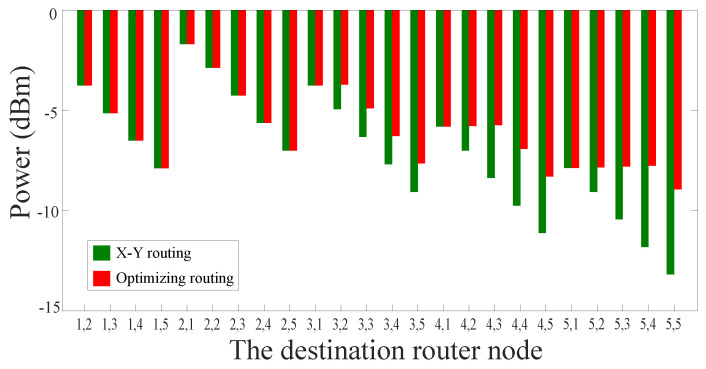
Signal power comparison between traditional routing algorithm and our optimization algorithm in a 5×5 mesh-based ONoC.

First of all, for a 5×5 mesh-based ONoC, when the optical signals travel from processor core (1, 1) to the other 24 processor cores, we analyze the power loss of the routing paths that were selected using the dimension-order routing algorithm. Subsequently, we utilize the novel algorithm, which was proposed by us, to select the optical routing paths with the minimum power loss. Figure 6 shows the optical signal power obtained by using the two algorithms. The transverse coordinates represent the destination routers, for example, ‘2, 1’ indicates that the optical signal travels from processor core (1, 1) to processor core (2, 1). According to Figure 6, it can be clearly observed that the simulation results are consistent with the results that we expected. When the source core and the destination core are located in the same X-axis or the same Y-axis, the routing paths chosen by the dimension-order routing algorithm are the same as those chosen by our algorithm.

For most other communication node pairs, the signal power of the optical links selected by our method is significantly better than that of the traditional routing paths, as can be seen from Figure 6. According to formula (19), we can know that our algorithm realizes an average of 16.82% reduction in power loss compared with the dimension-order routing algorithm. When the optical signal passes through the longest optical link, namely, the optical signal travels from the processor core (1, 1) to the processor core (5, 5), the signal power of the optical link selected by the dimension-order routing algorithm is −13.18 dBm, while the signal power of the optical link selected by our algorithm is −8.94 dBm.

**Figure 7:**
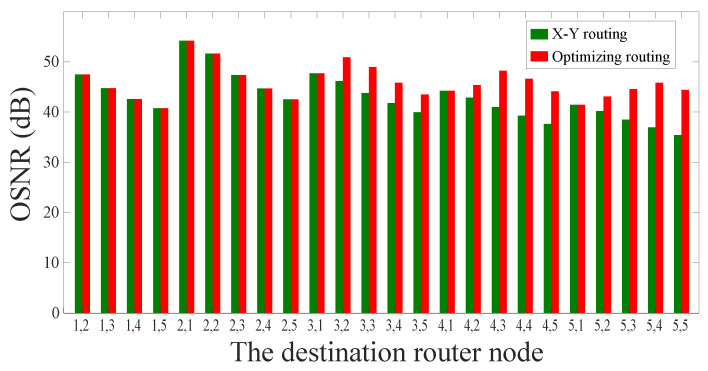
OSNR comparison between traditional routing algorithm and our optimization algorithm in a 5×5 mesh-based ONoC.

After that, we use the crosstalk model and the OSNR model proposed above in order to calculate the approximate worst crosstalk noise and the OSNR of the optical links. And we use the same models to analyze the traditional routing paths, and the OSNR comparison between the two routing algorithms is shown in Figure 7. According to the simulation results, we can obviously find that our method can not only ensure that the optical links have the lowest power loss, but also improve the OSNR of the routing paths. Specifically, the OSNR of the longest optical link selected by the dimension-order routing algorithm is 35.42 dB, while the OSNR of the longest optical link selected by our algorithm is 44.40 dB.

**Figure 8:**
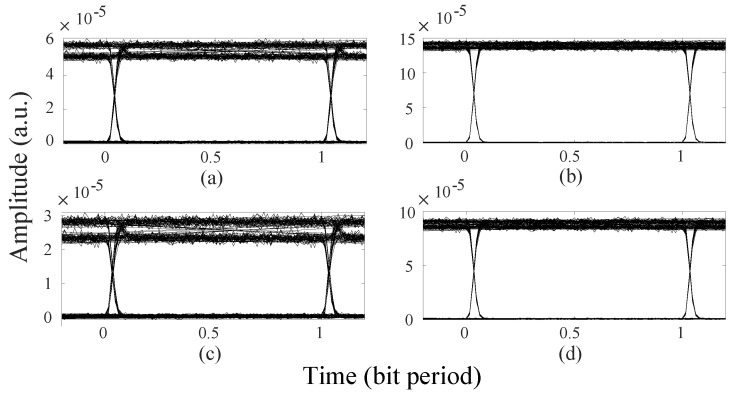
Four eye diagrams of different routing paths in mesh-based ONoC. (**a**) Processor core (1, 1) to processor core (5, 5): the eye diagram of the signal transmitted through the routing path selected by the traditional algorithm; (**b**) Processor core (1, 1) to processor core (5, 5): the eye diagram of the signal transmitted through the routing path selected by our optimization algorithm; (**c**) Processor core (1, 1) to processor core (6, 6): the eye diagram of the signal transmitted through the routing path selected by the traditional algorithm; (**d**) Processor core (1, 1) to processor core (6, 6): the eye diagram of the signal transmitted through the routing path selected by our optimization algorithm.

To be general, the eye diagram can reflect the influence of crosstalk noise intuitively. When the optical signal is transmitted from the processor core (1, 1) to the processor core (5, 5), the traditional algorithm and our algorithm are used to select the routing path, and the eye diagrams of the signals are shown in Figure 8a,b, respectively. It can be seen from the graph that the signal passes through the routing path that was selected by our algorithm has a higher eye height and a clearer eye diagram. Moreover, the Q factor can be a good measure of system performance. For the different optical links (from core (1, 1) to core (5, 5)) selected by the traditional algorithm and our algorithm, the maximum Q factor of the signal is 17.74 and 42.42, respectively.

**Figure 9:**
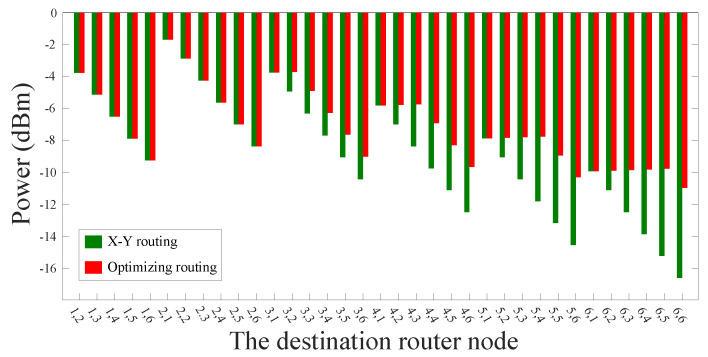
Signal power comparison between traditional routing algorithm and our optimization algorithm in a 6×6 mesh-based ONoC.

Subsequently, we expand the optical network size. For the 6×6 mesh-based ONoC, when the optical signals travel from the core (1, 1) to the other 35 cores, the optical signal power obtained by using the two algorithms are shown in Figure 9. We can find that the signal power of the optical links selected by our method is significantly better than that of the traditional routing paths for most communication node pairs. According to formula (19), our algorithm realizes an average of 21.19% reduction in power loss compared with the dimension-order routing algorithm. In addition, when the optical signal travels from the core (1, 1) to the core (6, 6), the signal power of the optical link selected by the dimension-order routing algorithm is −16.62 dBm, while the signal power of the optical link selected by our algorithm is −10.96 dBm. For the longest optical links selected by the traditional algorithm and our algorithm, the eye diagrams of the two signals are shown in Figure 8c,d, respectively, and the maximum Q factor of the signals is 11.55 and 33.13, respectively.

**Figure 10:**
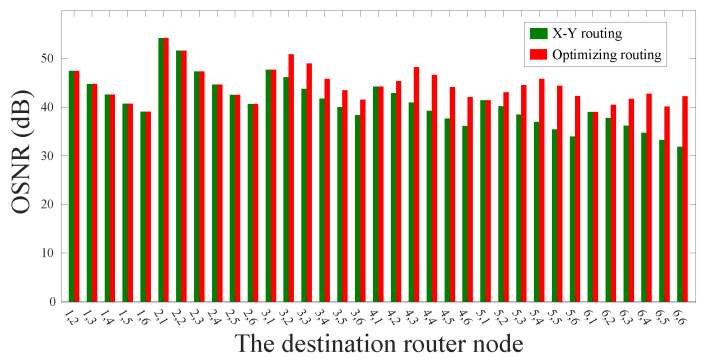
OSNR comparison between traditional routing algorithm and our optimization algorithm in a 6×6 mesh-based ONoC.

Afterwards, we calculate the approximate worst crosstalk noise and the OSNR of the optical links. Based on the same models, we analyze the traditional routing paths, and the OSNR comparison between the two routing algorithms is shown in Figure 10. For a 6×6 mesh-based ONoC, we take core (1, 1) as the source node and other nodes as the destination nodes. When compared with the optical links selected by the dimension-order routing algorithm, the OSNR of the optical links selected by our algorithm in this paper is higher for most communication node pairs. The OSNR of the longest optical link selected by the dimension-order routing algorithm is 31.83 dB, while the OSNR of the longest optical link selected by our algorithm is 42.25 dB.

**Figure 11:**
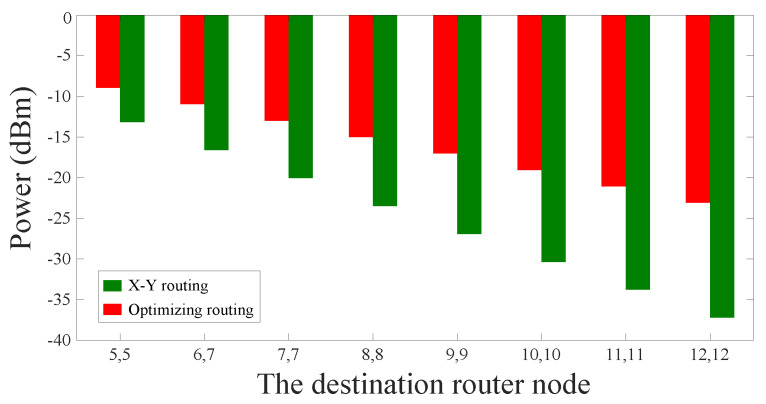
Signal power comparison of the longest optical links in different optical network sizes.

When the optical signal is transmitted from the processor core (1, 1) to the processor core (M,M) $M\in \left.\{5,6,7,8,9,10,11,12\right.\}$, the traditional algorithm and our algorithm are used in order to select the routing paths, and the optical signal power obtained by using the two algorithms is shown in Figure 11. From the simulation results, we can see that the signal power of the routing paths selected by our method is obviously better than that of the dimension-order routing paths. Additionally, we can find that the power loss of the optical links selected by our algorithm will be greatly optimized with the gradual increase of the scale of the optical network.

**Figure 12:**
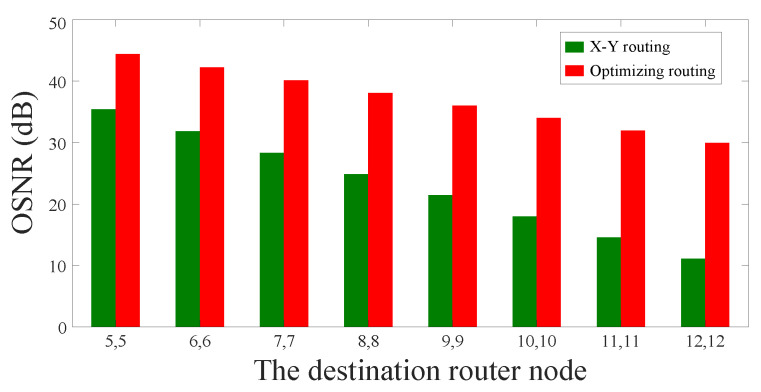
OSNR comparison of the longest optical links in different optical network sizes.

Subsequently, we use the calculation model proposed in Section 2 to calculate the crosstalk noise and the OSNR of the longest optical links obtained by the two routing algorithms. The OSNR comparison between the two algorithms is shown in Figure 12. We can find that the longest optical links selected by using our algorithm is significantly better than the longest optical links selected by using the dimension-order routing algorithm in terms of OSNR. Similar to the results of the power loss of the longest optical links, with the increase of the network scale, our algorithm will perform better in improving OSNR.

## 5. Conclusions

Power loss and OSNR are the key factors for measuring the performance of ONoCs. In this paper, we put forward a 5×5 all-pass optical router model for 2-D mesh-based ONoCs. Afterwards, a novel routing algorithm is proposed in order to select the routing paths with the minimum power loss based on the power loss model, the approximate worst crosstalk model, and the all-pass optical router model. We use the dimension-order routing algorithm as a comparison and take core (1, 1) as the source node and other cores as the destination nodes. The simulation results indicate that, when the size of ONoC is 5×5, using our algorithm can reduce the power loss by 16.82% on average, and the OSNR can also be improved. Furthermore, when the size of ONoC is 6×6, the power loss will be reduced by 21.19% on average, and the improvement of OSNR will be more obvious. In addition, when compared with the traditional algorithm, the optical paths selected by our algorithm have a higher Q factor and clearer eye diagrams. Furthermore, our algorithm will perform better in reducing the power loss and raising the OSNR with the gradual increase of the optical network scale.

## Figures and Tables

**Table 1:** The loss values and crosstalk coefficients.

Parameter	Value	Reference
$L_{b}$	−0.005 dB/90${}^{\circ }$	[28]
$L_{cr}$	−0.04 dB	[29]
$L_{pse,on}$	−0.5 dB	[30]
$L_{pse,off}$	−0.005 dB	[30]
$L_{cse,on}$	−0.5 dB	[12]
$L_{cse,off}$	−0.04 dB	[12]
$L_{p}$	−0.274 dB/cm	[12]
$C_{cr}$	−40 dB	[29]
$C_{pse,on}$	−25 dB	[30]
$C_{pse,off}$	−20 dB	[30]
